# (5,10,15,20-Tetra­phenyl­porphyrinato-κ^4^ *N*)zinc–18-crown-6 (1/1)

**DOI:** 10.1107/S1600536813018126

**Published:** 2013-07-10

**Authors:** Zouhour Denden, Leila Bel Haj Hassen, Mohamed Salah Belkhiria, Jean-Claude Daran, Habib Nasri

**Affiliations:** a Laboratoire de Physico-chimie des Matériaux, Université de Monastir, Faculté des Sciences de Monastir, Avenue de l’environnement, 5019 Monastir, Tunisia; b Laboratoire de Chimie de Coordination, CNRS UPR 8241, 205 route de Norbonne, 31077 Toulouse, Cedex 04, France

## Abstract

In the title compound, [Zn(C_44_H_28_N_4_)]·C_12_H_24_O_6_, the Zn^II^ ion lies on an inversion center and the asymmetric unit contains one half of a Zn(TPP) complex (TPP = 5,10,15,20-tetra­phenyl­porphyrin dianion) and one half of a centrosymmetric 18-crown-6 mol­ecule. The Zn(TPP) complex exhibits a nearly planar conformation of the porphyrin core [maximum deviation = 0.106 (2) Å] with an average Zn—N distance of 2.047 (2) Å. The title compound is considered as a one-dimensional polymer along [010], in which the Zn(TPP) moiety is linked to the closest O atoms of two symmetry-related 18-crown-6 mol­ecules with a Zn—O distance of 2.582 (1) Å, completing a distorted octahedral coordination environment of the metal ion. The chains are mainly sustained by weak C—H⋯π inter­actions. An ethyl­ene group of the 18-crown-6 mol­ecule is disordered over three sites with occupancies of 0.50, 0.25 and 0.25.

## Related literature

For related structures, see: Cheng & Scheidt (1995 ▶); Diskin-Posner *et al.* (1999 ▶); Ezzayani *et al.* (2013 ▶); Kojima *et al.* (2009 ▶); Kumar *et al.* (1997 ▶); Mansour *et al.* (2010 ▶); Ricard *et al.* (2001 ▶); Suijkerbuijk *et al.* (2007 ▶); Toumi *et al.* (2013 ▶). For the SIMU/ISOR restraints used in the refinement, see: McArdle (1995 ▶). For a description of the Cambridge Strcutural Database, see: Allen (2002 ▶). For the synthesis, see: Oberda *et al.* (2011 ▶).
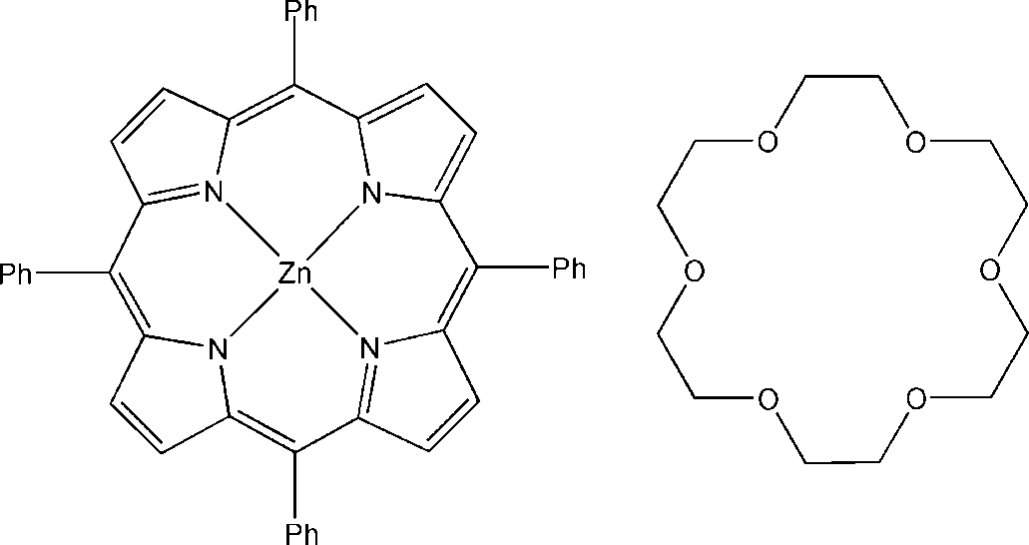


## Experimental

### 

#### Crystal data

[Zn(C_44_H_28_N_4_)]·C_12_H_24_O_6_
*M* *_r_* = 942.39
Triclinic, 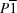

*a* = 10.2170 (3) Å
*b* = 11.1190 (4) Å
*c* = 11.8243 (3) Å
α = 104.384 (3)°
β = 105.912 (3)°
γ = 108.096 (3)°
*V* = 1143.23 (8) Å^3^
*Z* = 1
Mo *K*α radiation
μ = 0.60 mm^−1^
*T* = 180 K
0.48 × 0.45 × 0.33 mm

#### Data collection

Oxford Diffraction Xcalibur diffractometer
Absorption correction: multi-scan (*CrysAlis RED*; Oxford Diffraction, 2010 ▶) *T*_min_ = 0.918, *T*_max_ = 1.000
22963 measured reflections
4503 independent reflections
3774 reflections with *I* > 2σ(*I*)
*R*_int_ = 0.023

#### Refinement

*R*[*F* ^2^ > 2σ(*F* ^2^)] = 0.039
*wR*(*F* ^2^) = 0.108
*S* = 1.07
4503 reflections
310 parameters
30 restraints
H-atom parameters constrained
Δρ_max_ = 0.95 e Å^−3^
Δρ_min_ = −0.67 e Å^−3^

### 

Data collection: *CrysAlis CCD* (Oxford Diffraction, 2010 ▶); cell refinement: *CrysAlis RED* (Oxford Diffraction, 2010 ▶); data reduction: *CrysAlis RED*; program(s) used to solve structure: *SIR2004* (Burla *et al.*, 2005 ▶); program(s) used to refine structure: *SHELXL97* (Sheldrick, 2008 ▶); molecular graphics: *ORTEPIII* (Burnett & Johnson, 1996 ▶) and *ORTEP-3 for Windows* (Farrugia, 2012) ▶; software used to prepare material for publication: *WinGX* (Farrugia, 2012) ▶.

## Supplementary Material

Crystal structure: contains datablock(s) I. DOI: 10.1107/S1600536813018126/hy2633sup1.cif

Structure factors: contains datablock(s) I. DOI: 10.1107/S1600536813018126/hy2633Isup2.hkl

Additional supplementary materials:  crystallographic information; 3D view; checkCIF report

## Figures and Tables

**Table 1:** Hydrogen-bond geometry (Å, °) *Cg*1, *Cg*2 and *Cg*3 are the centroids of the N1/C1–C4, N2/C6–C9 and C11–C16 rings, respectively.

*D*—H⋯*A*	*D*—H	H⋯*A*	*D*⋯*A*	*D*—H⋯*A*
C15—H15⋯*Cg*1^i^	0.93	2.98	3.824 (2)	152
C20—H20⋯*Cg*3^ii^	0.93	2.84	3.746 (2)	164
C24—H24*A*⋯*Cg*2	0.97	2.73	3.686 (3)	167

Symmetry codes: (i) 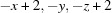; (ii) 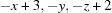.

**Fig. 1.**
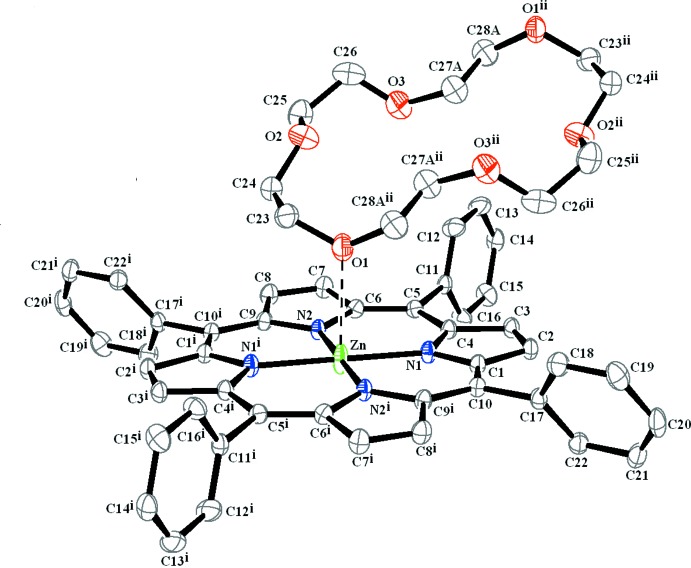
The molecular structure of the title compound. Displacement ellipsoids are drawn at the 40% probability level. H atoms have been omitted for clarity. [Symmtry codes: (i) -x+2, -y, -z+1; (ii) -x+2, -y-1, -z+1.]

**Fig. 2.**
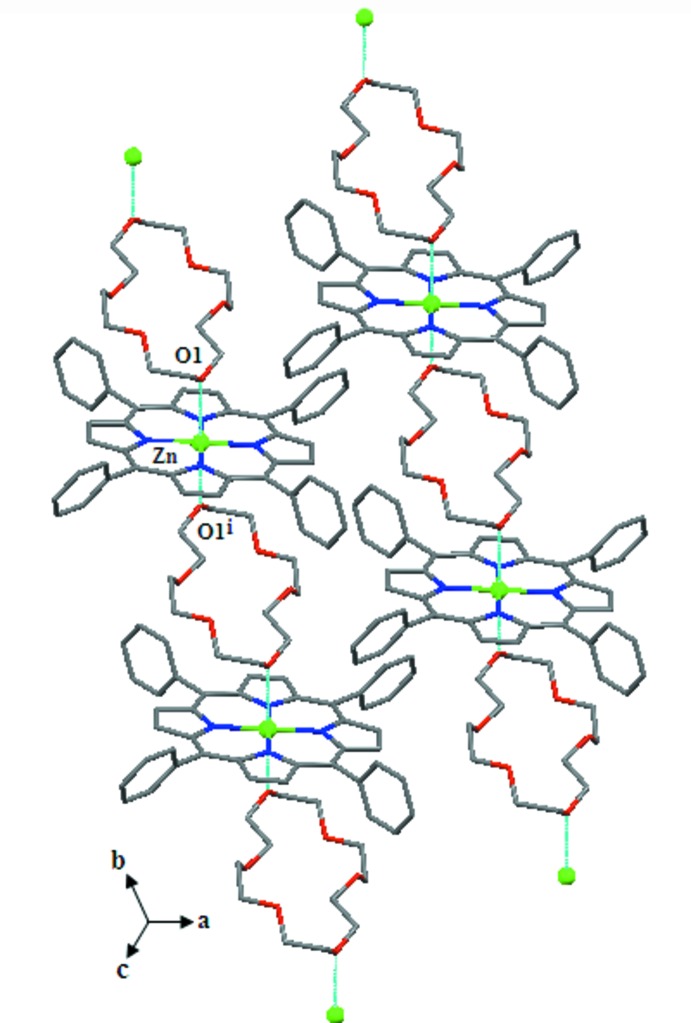
A drawing showing the one-dimensional structure of the title compound. H atoms have been omitted for clarity. [Symmetry code: (i) 2-x, -y, 1-z.]

## supplementary crystallographic information

### Comment

In continuation of our research on the crystal structures of metalloporphyrins resulting from the interactions of porphyrin complexes type [*M*^II^(TPP)] (TPP is a dianion of 5,10,15,20-tetraphenylporphyrin and *M* is a metal) with 18-crown-6 (18-C-6), we recently reported the molecular structures of three metalloporphyrines involving 18-C-6. The first one is (tetraphenylporphyrinato)cobalt(II)-18-C-6 with the formula [Co^II^(TPP)].(18-C-6) (Mansour *et al.*, 2010) and the second structure concerns the coordination complex diaqua(tetraphenylporphyrinato)magnesium(II)-18-C-6 with the formula [Mg(TPP)(H_2_O)_2_].(18-C-6) (Ezzayani *et al.*, 2013). The third metalloporphyrin-18-C-6 derivative is the [Cd(TPP)(H_2_O)].(18-C-6) species (Toumi *et al.*, 2013). By the other hand a search of Cambridge Structural Database (CSD, version 5.34; Allen, 2002) reveals only two zinc–porphyrin structures involving 18-C-6 molecules, with the same formula [Zn(TPP)(H_2_O)].(18-C-6) [CSD refcodes: ZOLXUT (Cheng & Scheidt, 1995), and XIYGAN (Diskin-Posner *et al.*, 1999)]. The average equatorial Zn—N distance equal to 2.071 (1) Å lies in the range [2.035 (2)–2.081 (5) Å] of related porphyrin species, *i.e.* [Zn(TPP)(H_2_O)_2_] (Suijkerbuijk *et al.*, 2007) and [Zn(TPP)(4-pyridinamine)] (Kojima *et al.*, 2009). In order to gain more insight into the interactions of 18-C-6 with zinc–porphyrin complexes, we report herein the synthesis and crystal structure of the title compound, [Zn(TPP)].(18-C-6).

In the title compound, two symmetry-related 18-C-6 molecules are weakly bonded to the Zn^II^ ion *via* the O1 atom with a distance of 2.582 (1) Å (Fig. 1). It is noteworthy that the Zn—O bond length values for metalloporphyrin type [Zn(porph)(O*R*)] (*R* = an alkyl or aryl group) are within the large range [2.147 (5)–2.708 (2) Å] [CSD refcodes: BOQPIG (Ricard *et al.*, 2001), and GETGER (Kumar *et al.*, 1997)]. Consequently, we can consider that for the title compound, the 18-C-6 molecule is weakly coordinated to the central metal. We noticed the striking resemblance of the title compound and the related compound [Co^II^(TPP)].(18-C-6): (i) the two structures are isomorphs and they have the space group *P*1 and the cell parameters are very close; (ii) the Co—O(18-C-6) distance [2.533 (2) Å] is quite close to the value of the title compound. The porphyrin core presents a nearly planar conformation with maximum and minimum deviations from the C_20_N_4_ least-squares plane of 0.106 (2) and 0.000 (2) Å for C10 and C3 atoms, respectively, while the Zn^II^ ion is at 0.05 (1) Å from the plane.

The crystal structure of the title compound resembles to one-dimensional coordination polymer, where each [Zn(TPP)] moiety is linked to two symmetry-related 18-C-6 molecules through the O1 and O1^i^ atoms (symmetry code: (i) 2-x, -y, 1-z) (Fig. 2). These chains are mainly sustained by weak C—H···π interactions (Table 1).

### Experimental

The reaction of the Zn(TPP) complex (15 mg, 0.022 mmol) (Oberda *et al.*, 2011) with an excess of 18-C-6 (80 mg, 0.302 mmol) in 15 ml of chlorobenzene at room temperature yielded after two hours a red-purple solution. Crystals of the title compound were obtained by diffusion of hexane into the chlorobenzene solution.

### Refinement

All H atoms were placed in geometrically idealized positions and refined as riding atoms, with C—H = 0.93 (aromatic) and 0.97 (CH_2_) Å and with *U*_iso_(H) = 1.2*U*_eq_(C). An ethylene group of the 18-C-6 molecule is disordered over three sites with occupancies of 0.50, 0.25 and 0.25. The anisotropic ellipsoids of the atoms O3, C27A and C28A of the 18-C-6 molecule were elongated, so the SIMU/ISOR restraints (McArdle, 1995) and EADP constraint commands in the *SHELXL97* software (Sheldrick, 2008) were used .

### Crystal data

**Table:**

[Zn(C_44_H_28_N_4_)]·C_12_H_24_O_6_	*Z* = 1
*M**_r_* = 942.39	*F*(000) = 494
Triclinic, *P*1	*D*_x_ = 1.369 Mg m^−^^3^
Hall symbol: -P 1	Mo *K*α radiation, λ = 0.71073 Å
*a* = 10.2170 (3) Å	Cell parameters from 13909 reflections
*b* = 11.1190 (4) Å	θ = 3.3–29.1°
*c* = 11.8243 (3) Å	µ = 0.60 mm^−^^1^
α = 104.384 (3)°	*T* = 180 K
β = 105.912 (3)°	Block, violet
γ = 108.096 (3)°	0.48 × 0.45 × 0.33 mm
*V* = 1143.23 (8) Å^3^	

### Data collection

**Table:**

Oxford Diffraction Xcalibur diffractometer	4503 independent reflections
Radiation source: fine-focus sealed tube	3774 reflections with *I* > 2σ(*I*)
Graphite monochromator	*R*_int_ = 0.023
Detector resolution: 16.1978 pixels mm^-1^	θ_max_ = 26.0°, θ_min_ = 3.3°
ω scans	*h* = −12→12
Absorption correction: multi-scan (*CrysAlis RED*; Oxford Diffraction, 2010)	*k* = −13→13
*T*_min_ = 0.918, *T*_max_ = 1.000	*l* = −14→14
22963 measured reflections	

### Refinement

**Table:**

Refinement on *F*^2^	Primary atom site location: structure-invariant direct methods
Least-squares matrix: full	Secondary atom site location: difference Fourier map
*R*[*F*^2^ > 2σ(*F*^2^)] = 0.039	Hydrogen site location: inferred from neighbouring sites
*wR*(*F*^2^) = 0.108	H-atom parameters constrained
*S* = 1.07	*w* = 1/[σ^2^(*F*_o_^2^) + (0.0591*P*)^2^ + 0.5621*P*] where *P* = (*F*_o_^2^ + 2*F*_c_^2^)/3
4503 reflections	(Δ/σ)_max_ < 0.001
310 parameters	Δρ_max_ = 0.95 e Å^−^^3^
30 restraints	Δρ_min_ = −0.67 e Å^−^^3^

### Special details

**Table:**

Geometry. All s.u.'s (except the s.u. in the dihedral angle between two l.s. planes) are estimated using the full covariance matrix. The cell s.u.'s are taken into account individually in the estimation of s.u.'s in distances, angles and torsion angles; correlations between s.u.'s in cell parameters are only used when they are defined by crystal symmetry. An approximate (isotropic) treatment of cell s.u.'s is used for estimating s.u.'s involving l.s. planes.
Refinement. Refinement of *F*^2^ against ALL reflections. The weighted *R*-factor *wR* and goodness of fit *S* are based on *F*^2^, conventional *R*-factors *R* are based on *F*, with *F* set to zero for negative *F*^2^. The threshold expression of *F*^2^ > 2σ(*F*^2^) is used only for calculating *R*-factors(gt) *etc*. and is not relevant to the choice of reflections for refinement. *R*-factors based on *F*^2^ are statistically about twice as large as those based on *F*, and *R*- factors based on ALL data will be even larger.

### Fractional atomic coordinates and isotropic or equivalent isotropic displacement parameters (Å^2^)

**Table:**

	*x*	*y*	*z*	*U*_iso_*/*U*_eq_	Occ. (<1)
Zn	1.0000	0.0000	0.5000	0.03274 (14)	
N1	1.11054 (18)	−0.01373 (18)	0.66650 (15)	0.0230 (4)	
N2	0.79951 (17)	−0.10495 (17)	0.50470 (15)	0.0213 (3)	
C1	1.2617 (2)	0.0346 (2)	0.72913 (18)	0.0219 (4)	
C2	1.2919 (2)	−0.0065 (2)	0.83665 (19)	0.0248 (4)	
H2	1.3854	0.0140	0.8949	0.030*	
C3	1.1598 (2)	−0.0801 (2)	0.83723 (18)	0.0243 (4)	
H3	1.1450	−0.1199	0.8958	0.029*	
C4	1.0454 (2)	−0.0857 (2)	0.72994 (18)	0.0215 (4)	
C5	0.8921 (2)	−0.1589 (2)	0.69362 (18)	0.0214 (4)	
C6	0.7795 (2)	−0.1682 (2)	0.58835 (18)	0.0217 (4)	
C7	0.6231 (2)	−0.2468 (2)	0.5519 (2)	0.0274 (5)	
H7	0.5805	−0.2996	0.5927	0.033*	
C8	0.5497 (2)	−0.2298 (2)	0.4477 (2)	0.0270 (4)	
H8	0.4471	−0.2677	0.4038	0.032*	
C9	0.6606 (2)	−0.1415 (2)	0.41709 (18)	0.0215 (4)	
C10	1.3700 (2)	0.1076 (2)	0.69132 (18)	0.0221 (4)	
C11	0.8443 (2)	−0.2378 (2)	0.77232 (18)	0.0218 (4)	
C12	0.8211 (3)	−0.3733 (2)	0.7389 (2)	0.0355 (5)	
H12	0.8369	−0.4151	0.6684	0.043*	
C13	0.7744 (3)	−0.4473 (2)	0.8098 (2)	0.0382 (6)	
H13	0.7593	−0.5383	0.7867	0.046*	
C14	0.7504 (2)	−0.3870 (2)	0.9138 (2)	0.0318 (5)	
H14	0.7190	−0.4368	0.9611	0.038*	
C15	0.7733 (3)	−0.2519 (2)	0.9478 (2)	0.0338 (5)	
H15	0.7569	−0.2105	1.0181	0.041*	
C16	0.8207 (2)	−0.1776 (2)	0.8774 (2)	0.0288 (5)	
H16	0.8366	−0.0864	0.9013	0.035*	
C17	1.5297 (2)	0.1524 (2)	0.77206 (18)	0.0228 (4)	
C18	1.6208 (2)	0.1016 (2)	0.7256 (2)	0.0303 (5)	
H18	1.5813	0.0383	0.6437	0.036*	
C19	1.7692 (3)	0.1441 (3)	0.7995 (2)	0.0373 (5)	
H19	1.8287	0.1094	0.7672	0.045*	
C20	1.8297 (2)	0.2382 (3)	0.9214 (2)	0.0366 (6)	
H20	1.9298	0.2674	0.9706	0.044*	
C21	1.7406 (2)	0.2884 (2)	0.9696 (2)	0.0337 (5)	
H21	1.7805	0.3507	1.0519	0.040*	
C22	1.59147 (13)	0.24577 (12)	0.89529 (11)	0.0270 (4)	
H22	1.5321	0.2801	0.9283	0.032*	
O1	0.99283 (13)	−0.23197 (12)	0.38069 (11)	0.0414 (4)	
C23	0.88485 (13)	−0.30609 (12)	0.25589 (11)	0.0415 (6)	
H23A	0.9247	−0.3582	0.2072	0.050*	
H23B	0.8669	−0.2424	0.2171	0.050*	
C24	0.7397 (3)	−0.4009 (3)	0.2491 (2)	0.0423 (6)	
H24A	0.7125	−0.3563	0.3149	0.051*	
H24B	0.6622	−0.4252	0.1681	0.051*	
O2	0.7523 (2)	−0.51824 (18)	0.26441 (18)	0.0450 (4)	
C25	0.6304 (3)	−0.6046 (3)	0.2816 (3)	0.0473 (6)	
H25A	0.5470	−0.6566	0.2012	0.057*	
H25B	0.5991	−0.5509	0.3380	0.057*	
C26	0.6787 (3)	−0.6981 (3)	0.3367 (3)	0.0510 (7)	
H26A	0.5923	−0.7734	0.3282	0.061*	
H26B	0.7329	−0.7344	0.2921	0.061*	
O3	0.7692 (2)	−0.6263 (2)	0.4630 (2)	0.0602 (5)	
C27A	0.9180 (7)	−0.6327 (7)	0.5200 (6)	0.0541 (7)	0.50
H27A	0.9569	−0.6576	0.4558	0.065*	0.50
H27B	0.9902	−0.5461	0.5848	0.065*	0.50
C28A	0.8820 (7)	−0.7408 (7)	0.5755 (6)	0.0492 (8)	0.50
H28A	0.8521	−0.7103	0.6447	0.059*	0.50
H28B	0.7999	−0.8229	0.5118	0.059*	0.50
C27B	0.8033 (15)	−0.7226 (13)	0.5251 (14)	0.0541 (7)	0.25
H27C	0.7839	−0.7096	0.6022	0.065*	0.25
H27D	0.7453	−0.8165	0.4685	0.065*	0.25
C28B	0.9557 (13)	−0.6854 (13)	0.5508 (13)	0.0492 (8)	0.25
H28C	1.0109	−0.5895	0.6021	0.059*	0.25
H28D	0.9724	−0.7018	0.4725	0.059*	0.25
C27C	0.8191 (15)	−0.7395 (13)	0.4715 (13)	0.0541 (7)	0.25
H27E	0.7387	−0.8215	0.4604	0.065*	0.25
H27F	0.8663	−0.7600	0.4127	0.065*	0.25
C28C	0.9287 (15)	−0.6667 (14)	0.6059 (12)	0.0492 (8)	0.25
H28E	0.8790	−0.6567	0.6645	0.059*	0.25
H28F	0.9991	−0.5779	0.6184	0.059*	0.25

### Atomic displacement parameters (Å^2^)

**Table:**

	*U*^11^	*U*^22^	*U*^33^	*U*^12^	*U*^13^	*U*^23^
Zn	0.01801 (19)	0.0560 (3)	0.0266 (2)	0.00947 (17)	0.00743 (14)	0.02734 (18)
N1	0.0178 (8)	0.0310 (9)	0.0204 (8)	0.0074 (7)	0.0065 (7)	0.0138 (7)
N2	0.0182 (8)	0.0277 (9)	0.0180 (8)	0.0078 (7)	0.0058 (6)	0.0116 (7)
C1	0.0206 (9)	0.0250 (10)	0.0183 (9)	0.0085 (8)	0.0049 (8)	0.0089 (8)
C2	0.0232 (10)	0.0294 (11)	0.0200 (10)	0.0106 (9)	0.0040 (8)	0.0110 (8)
C3	0.0257 (10)	0.0285 (11)	0.0191 (9)	0.0102 (9)	0.0066 (8)	0.0125 (8)
C4	0.0234 (10)	0.0252 (10)	0.0176 (9)	0.0098 (8)	0.0080 (8)	0.0103 (8)
C5	0.0249 (10)	0.0227 (10)	0.0185 (9)	0.0091 (8)	0.0105 (8)	0.0091 (8)
C6	0.0217 (10)	0.0245 (10)	0.0198 (9)	0.0076 (8)	0.0101 (8)	0.0096 (8)
C7	0.0226 (10)	0.0338 (12)	0.0268 (11)	0.0072 (9)	0.0112 (9)	0.0158 (9)
C8	0.0188 (10)	0.0318 (11)	0.0272 (11)	0.0057 (9)	0.0078 (8)	0.0127 (9)
C9	0.0184 (9)	0.0255 (10)	0.0198 (9)	0.0077 (8)	0.0070 (8)	0.0091 (8)
C10	0.0199 (9)	0.0244 (10)	0.0201 (9)	0.0088 (8)	0.0054 (8)	0.0082 (8)
C11	0.0181 (9)	0.0258 (10)	0.0194 (9)	0.0054 (8)	0.0055 (8)	0.0113 (8)
C12	0.0522 (15)	0.0290 (12)	0.0245 (11)	0.0139 (11)	0.0175 (10)	0.0089 (9)
C13	0.0504 (15)	0.0233 (11)	0.0313 (12)	0.0065 (11)	0.0106 (11)	0.0109 (10)
C14	0.0276 (11)	0.0368 (13)	0.0288 (11)	0.0050 (10)	0.0084 (9)	0.0213 (10)
C15	0.0431 (13)	0.0442 (14)	0.0296 (11)	0.0230 (11)	0.0229 (10)	0.0214 (10)
C16	0.0379 (12)	0.0288 (11)	0.0281 (11)	0.0163 (10)	0.0170 (9)	0.0152 (9)
C17	0.0202 (10)	0.0256 (10)	0.0225 (10)	0.0072 (8)	0.0060 (8)	0.0137 (8)
C18	0.0275 (11)	0.0339 (12)	0.0295 (11)	0.0125 (10)	0.0107 (9)	0.0116 (9)
C19	0.0259 (11)	0.0465 (14)	0.0495 (14)	0.0193 (11)	0.0167 (11)	0.0254 (12)
C20	0.0182 (10)	0.0450 (14)	0.0424 (13)	0.0069 (10)	0.0018 (9)	0.0274 (11)
C21	0.0298 (12)	0.0333 (12)	0.0255 (11)	0.0041 (10)	0.0001 (9)	0.0134 (9)
C22	0.0256 (10)	0.0277 (11)	0.0254 (10)	0.0092 (9)	0.0071 (8)	0.0110 (9)
O1	0.0372 (9)	0.0465 (10)	0.0370 (9)	0.0164 (8)	0.0067 (7)	0.0185 (8)
C23	0.0559 (16)	0.0368 (13)	0.0301 (12)	0.0170 (12)	0.0140 (11)	0.0145 (10)
C24	0.0449 (14)	0.0412 (14)	0.0350 (13)	0.0208 (12)	0.0062 (11)	0.0106 (11)
O2	0.0465 (10)	0.0412 (10)	0.0627 (12)	0.0218 (9)	0.0329 (9)	0.0252 (9)
C25	0.0296 (12)	0.0583 (17)	0.0482 (15)	0.0101 (12)	0.0137 (11)	0.0212 (13)
C26	0.0595 (18)	0.0364 (14)	0.0672 (19)	0.0201 (13)	0.0383 (16)	0.0188 (13)
O3	0.0635 (8)	0.0611 (8)	0.0590 (8)	0.0356 (6)	0.0160 (6)	0.0219 (6)
C27A	0.0561 (9)	0.0546 (9)	0.0546 (9)	0.0271 (7)	0.0179 (7)	0.0225 (7)
C28A	0.0508 (10)	0.0514 (10)	0.0503 (10)	0.0242 (8)	0.0202 (7)	0.0213 (8)
C27B	0.0561 (9)	0.0546 (9)	0.0546 (9)	0.0271 (7)	0.0179 (7)	0.0225 (7)
C28B	0.0508 (10)	0.0514 (10)	0.0503 (10)	0.0242 (8)	0.0202 (7)	0.0213 (8)
C27C	0.0561 (9)	0.0546 (9)	0.0546 (9)	0.0271 (7)	0.0179 (7)	0.0225 (7)
C28C	0.0508 (10)	0.0514 (10)	0.0503 (10)	0.0242 (8)	0.0202 (7)	0.0213 (8)

### Geometric parameters (Å, º)

**Table:**

Zn—N2	2.0421 (16)	C21—C22	1.389 (2)
Zn—N1	2.0524 (16)	C21—H21	0.9300
N1—C1	1.372 (2)	C22—H22	0.9300
N1—C4	1.374 (2)	O1—C28A^ii^	1.397 (6)
N2—C9	1.371 (2)	O1—C23	1.4185
N2—C6	1.372 (2)	O1—C28B^ii^	1.487 (13)
C1—C10	1.405 (3)	O1—C28C^ii^	1.587 (12)
C1—C2	1.443 (3)	C23—C24	1.496 (3)
C2—C3	1.347 (3)	C23—H23A	0.9700
C2—H2	0.9300	C23—H23B	0.9700
C3—C4	1.443 (3)	C24—O2	1.400 (3)
C3—H3	0.9300	C24—H24A	0.9700
C4—C5	1.402 (3)	C24—H24B	0.9700
C5—C6	1.400 (3)	O2—C25	1.417 (3)
C5—C11	1.504 (3)	C25—C26	1.492 (4)
C6—C7	1.439 (3)	C25—H25A	0.9700
C7—C8	1.346 (3)	C25—H25B	0.9700
C7—H7	0.9300	C26—O3	1.384 (4)
C8—C9	1.445 (3)	C26—H26A	0.9700
C8—H8	0.9300	C26—H26B	0.9700
C9—C10^i^	1.406 (3)	O3—C27C	1.513 (12)
C10—C9^i^	1.406 (3)	O3—C27B	1.513 (13)
C10—C17	1.493 (3)	O3—C27A	1.517 (6)
C11—C16	1.379 (3)	C27A—C28A	1.504 (9)
C11—C12	1.384 (3)	C27A—H27A	0.9700
C12—C13	1.388 (3)	C27A—H27B	0.9700
C12—H12	0.9300	C28A—O1^ii^	1.397 (6)
C13—C14	1.370 (3)	C28A—H28A	0.9700
C13—H13	0.9300	C28A—H28B	0.9700
C14—C15	1.380 (3)	C27B—C28B	1.403 (18)
C14—H14	0.9300	C27B—H27C	0.9700
C15—C16	1.386 (3)	C27B—H27D	0.9700
C15—H15	0.9300	C28B—O1^ii^	1.487 (12)
C16—H16	0.9300	C28B—H28C	0.9700
C17—C22	1.392 (2)	C28B—H28D	0.9700
C17—C18	1.393 (3)	C27C—C28C	1.502 (18)
C18—C19	1.382 (3)	C27C—H27E	0.9700
C18—H18	0.9300	C27C—H27F	0.9700
C19—C20	1.383 (4)	C28C—O1^ii^	1.587 (12)
C19—H19	0.9300	C28C—H28E	0.9700
C20—C21	1.381 (4)	C28C—H28F	0.9700
C20—H20	0.9300		
			
N2—Zn—N2^i^	180.0	C20—C21—H21	120.0
N2—Zn—N1^i^	89.28 (6)	C22—C21—H21	120.0
N2^i^—Zn—N1^i^	90.72 (6)	C21—C22—C17	120.83 (16)
N2—Zn—N1	90.72 (6)	C21—C22—H22	119.6
N2^i^—Zn—N1	89.28 (6)	C17—C22—H22	119.6
N1^i^—Zn—N1	180.0	C28A^ii^—O1—C23	121.4 (3)
C1—N1—C4	106.75 (16)	C23—O1—C28B^ii^	115.6 (5)
C1—N1—Zn	127.32 (13)	C23—O1—C28C^ii^	99.7 (5)
C4—N1—Zn	125.65 (13)	O1—C23—C24	113.75 (11)
C9—N2—C6	106.67 (16)	O1—C23—H23A	108.8
C9—N2—Zn	127.28 (13)	C24—C23—H23A	108.8
C6—N2—Zn	125.51 (13)	O1—C23—H23B	108.8
N1—C1—C10	125.32 (18)	C24—C23—H23B	108.8
N1—C1—C2	109.25 (17)	H23A—C23—H23B	107.7
C10—C1—C2	125.37 (18)	O2—C24—C23	109.77 (19)
C3—C2—C1	107.45 (17)	O2—C24—H24A	109.7
C3—C2—H2	126.3	C23—C24—H24A	109.7
C1—C2—H2	126.3	O2—C24—H24B	109.7
C2—C3—C4	107.10 (17)	C23—C24—H24B	109.7
C2—C3—H3	126.5	H24A—C24—H24B	108.2
C4—C3—H3	126.5	C24—O2—C25	114.6 (2)
N1—C4—C5	125.70 (17)	O2—C25—C26	108.3 (2)
N1—C4—C3	109.43 (17)	O2—C25—H25A	110.0
C5—C4—C3	124.81 (18)	C26—C25—H25A	110.0
C6—C5—C4	125.74 (18)	O2—C25—H25B	110.0
C6—C5—C11	116.99 (17)	C26—C25—H25B	110.0
C4—C5—C11	117.24 (17)	H25A—C25—H25B	108.4
N2—C6—C5	126.10 (18)	O3—C26—C25	108.8 (2)
N2—C6—C7	109.46 (17)	O3—C26—H26A	109.9
C5—C6—C7	124.44 (18)	C25—C26—H26A	109.9
C8—C7—C6	107.41 (18)	O3—C26—H26B	109.9
C8—C7—H7	126.3	C25—C26—H26B	109.9
C6—C7—H7	126.3	H26A—C26—H26B	108.3
C7—C8—C9	107.03 (18)	C26—O3—C27C	91.8 (5)
C7—C8—H8	126.5	C26—O3—C27B	109.8 (6)
C9—C8—H8	126.5	C26—O3—C27A	121.5 (3)
N2—C9—C10^i^	125.72 (18)	C28A—C27A—O3	103.6 (5)
N2—C9—C8	109.42 (17)	C28A—C27A—H27A	111.0
C10^i^—C9—C8	124.69 (18)	O3—C27A—H27A	111.0
C1—C10—C9^i^	124.99 (18)	C28A—C27A—H27B	111.0
C1—C10—C17	117.65 (17)	O3—C27A—H27B	111.0
C9^i^—C10—C17	117.36 (18)	H27A—C27A—H27B	109.0
C16—C11—C12	118.84 (19)	O1^ii^—C28A—C27A	109.5 (5)
C16—C11—C5	120.98 (18)	O1^ii^—C28A—H28A	109.8
C12—C11—C5	120.17 (18)	C27A—C28A—H28A	109.8
C11—C12—C13	120.5 (2)	O1^ii^—C28A—H28B	109.8
C11—C12—H12	119.8	C27A—C28A—H28B	109.8
C13—C12—H12	119.8	H28A—C28A—H28B	108.2
C14—C13—C12	120.3 (2)	C28B—C27B—O3	102.5 (10)
C14—C13—H13	119.8	C28B—C27B—H27C	111.3
C12—C13—H13	119.8	O3—C27B—H27C	111.3
C13—C14—C15	119.6 (2)	C28B—C27B—H27D	111.3
C13—C14—H14	120.2	O3—C27B—H27D	111.3
C15—C14—H14	120.2	H27C—C27B—H27D	109.2
C14—C15—C16	120.2 (2)	C27B—C28B—O1^ii^	108.4 (10)
C14—C15—H15	119.9	C27B—C28B—H28C	110.0
C16—C15—H15	119.9	O1^ii^—C28B—H28C	110.0
C11—C16—C15	120.6 (2)	C27B—C28B—H28D	110.0
C11—C16—H16	119.7	O1^ii^—C28B—H28D	110.0
C15—C16—H16	119.7	H28C—C28B—H28D	108.4
C22—C17—C18	118.35 (17)	C28C—C27C—O3	96.2 (9)
C22—C17—C10	121.02 (17)	C28C—C27C—H27E	112.5
C18—C17—C10	120.63 (18)	O3—C27C—H27E	112.5
C19—C18—C17	120.8 (2)	C28C—C27C—H27F	112.5
C19—C18—H18	119.6	O3—C27C—H27F	112.5
C17—C18—H18	119.6	H27E—C27C—H27F	110.0
C18—C19—C20	120.3 (2)	C27C—C28C—O1^ii^	100.7 (9)
C18—C19—H19	119.9	C27C—C28C—H28E	111.6
C20—C19—H19	119.9	O1^ii^—C28C—H28E	111.6
C21—C20—C19	119.7 (2)	C27C—C28C—H28F	111.6
C21—C20—H20	120.1	O1^ii^—C28C—H28F	111.6
C19—C20—H20	120.1	H28E—C28C—H28F	109.4
C20—C21—C22	120.0 (2)		

Symmetry codes: (i) −*x*+2, −*y*, −*z*+1; (ii) −*x*+2, −*y*−1, −*z*+1.

### Hydrogen-bond geometry (Å, º)

Cg1, Cg2 and Cg3 are the centroids of the N1/C1–C4, N2/C6–C9 and C11–C16 rings, respectively.

**Table:**

*D*—H···*A*	*D*—H	H···*A*	*D*···*A*	*D*—H···*A*
C15—H15···*Cg*1^iii^	0.93	2.98	3.824 (2)	152
C20—H20···*Cg*3^iv^	0.93	2.84	3.746 (2)	164
C24—H24*A*···*Cg*2	0.97	2.73	3.686 (3)	167

Symmetry codes: (iii) −*x*+2, −*y*, −*z*+2; (iv) −*x*+3, −*y*, −*z*+2.
